# Vitamin-D Deficiency As a Potential Environmental Risk Factor in Multiple Sclerosis, Schizophrenia, and Autism

**DOI:** 10.3389/fpsyt.2017.00047

**Published:** 2017-03-27

**Authors:** Eva Kočovská, Fiona Gaughran, Amir Krivoy, Ute-Christiane Meier

**Affiliations:** ^1^Centre for Primary Care and Public Health, Barts and The London School of Medicine and Dentistry, Blizard Institute, Queen Mary University of London, London, UK; ^2^Department of Psychosis Studies, King’s College London, Institute of Psychiatry, Psychology and Neuroscience, National Psychosis Service, South London and Maudlsey NHS Foundation Trust, London, UK; ^3^Neuroinflammation and Psychoimmunology Group, Department of Neuroscience and Trauma, Barts and The London School of Medicine and Dentistry, Blizard Institute, Queen Mary University of London, London, UK

**Keywords:** vitamin-D, multiple sclerosis, schizophrenia, autism, immunity, microbiome

## Abstract

In this short review, we want to summarize the current findings on the role of vitamin-D in multiple sclerosis (MS), schizophrenia, and autism. Many studies have highlighted hypovitaminosis-D as a potential environmental risk factor for a variety of conditions such as MS, asthma, cardiovascular disease, and, more recently, psychiatric diseases. However, whether hypovitaminosis-D is a potential causative factor for the development or activity in these conditions or whether hypovitaminosis-D may be due to increased vitamin-D consumption by an activated immune system (reverse causation) is the focus of intense research. Here, we will discuss current evidence exploring the role of vitamin-D in MS, schizophrenia, and autism and its impact on adaptive and innate immunity, antimicrobial defense, the microbiome, neuroinflammation, behavior, and neurogenesis. More work is needed to gain insight into its role in the underlying pathophysiology of these conditions as it may offer attractive means of intervention and prevention.

## Vitamin-D Biology

Vitamin-D is a member of the family of steroid hormones together with sex hormones, retinoid, and cortisol. Vitamin-D has pleiotropic functions and plays an important role not only in calcium homeostasis and bone metabolism but also in regulating immune responses and hormonal and metabolic processes. Furthermore, it influences neurotropic and neuroprotective processes in the brain and may also impact on neurotransmission and synaptic plasticity (1–4). Its receptor has been found expressed in most tissues and organs (5).

**Figure 1:**
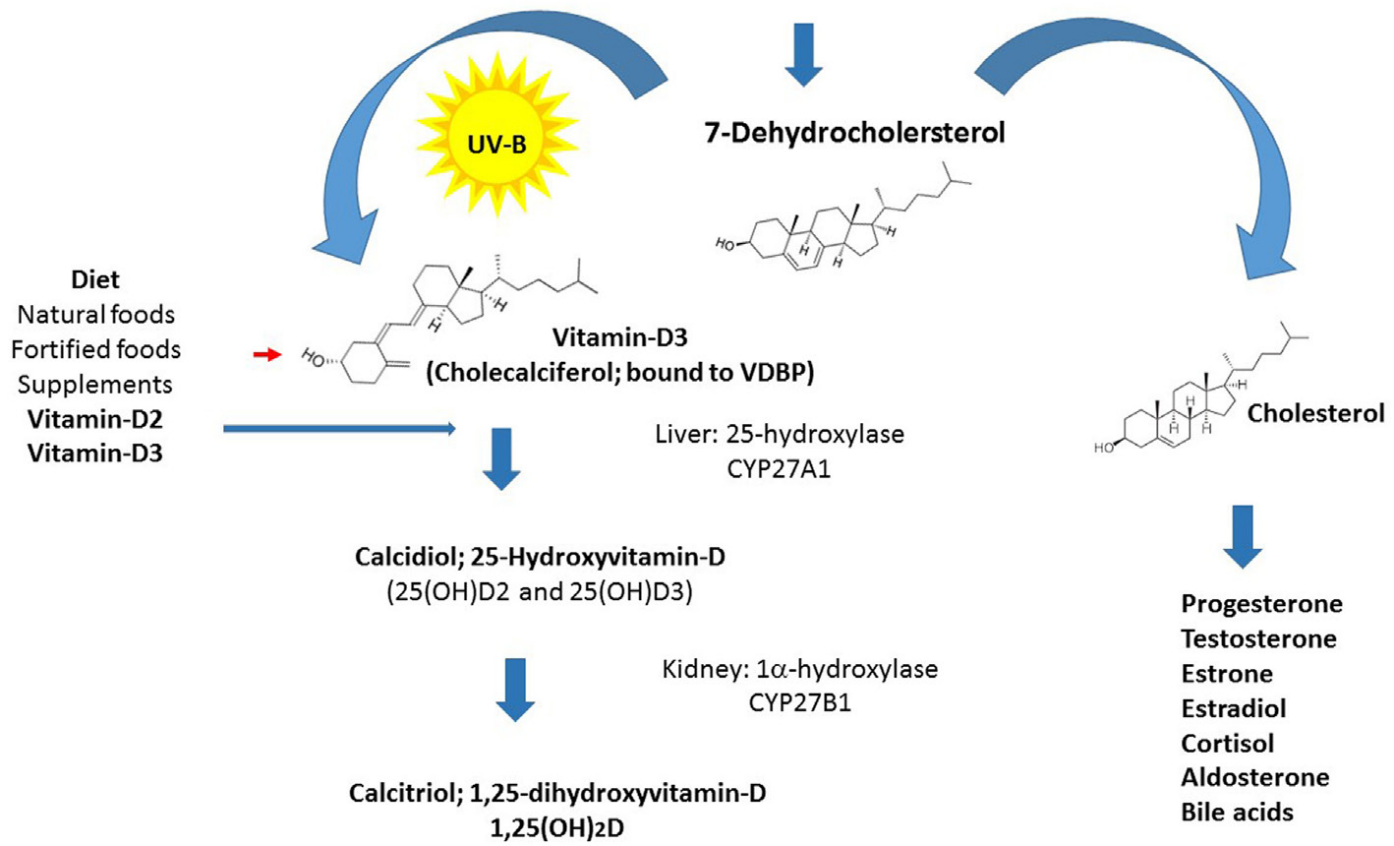
**Vitamin-D synthesis: schematic diagram of vitamin-D synthesis**.

Vitamin-D is the only steroid hormone not synthesized from cholesterol, and this exclusive metabolic pathway distinguishes it from all other steroid hormones and suggests important functions (Figure 1). Life on earth began approximately 3.5 billion years ago, and vitamin-D became pivotal to the evolution of humankind. Through its role in calcium homeostasis and the endocrine system, vitamin-D played an important part in our movement from the ocean to land and in the subsequent development of the calcified skeleton of the terrestrial *Homo sapiens* (6–8). Human life started in surroundings abundant in ultraviolet B (UVB) rays, and to this day, people living in this environment have average vitamin-D levels around 115 nmol/L (9). Our subsequent settlement in the northern hemisphere was accompanied by skin color changes to improve light absorption. During the past 200 years, our lifestyle changed dramatically and occurs mainly indoors, culminating in sun avoidance education, and the introduction of sun blockers over the last 50 years, leading to widespread hypovitaminosis-D (10).

Vitamin-D consists of two forms, vitamin-D_3_ and vitamin-D_2_, which are both biologically inert. The biosynthesis of the active form of vitamin-D (calcitriol) starts from its prime precursor 7-dehydrocholesterol and undergoes the key photochemical electrocyclization in the skin by irradiation with UVB light (at 290–315 nm), producing an intermediate that is spontaneously converted into vitamin-D_3_ (calciferol or cholecalciferol). Cholecalciferol is then transported to the liver, where it is enzymatically hydroxylated in the side chain at position 25 (the number refers to the position in the molecule, which elicits its highly specific biological properties) to produce calcidiol (25-hydroxyvitamin-D_3_). Vitamin-D_2_ and vitamin-D_3_ absorbed from the intestine are also metabolized in the liver. Calcidiol is subsequently converted to 1,25-dihydroxyvitamin-D [1,25(OH)_2_D] also known as calcitriol in the kidney by the action of the 1α-hydroxylase enzyme (11–13). Enzyme levels are controlled by the parathyroid hormone, whose secretion is in turn triggered by low concentrations of calcium or phosphate (14, 15). The latter enzymatic hydroxylation reaction, producing calcitriol, has also been found to occur in lymphocytes and in the brain in microglia and probably in other locations (16). The half-life of calcidiol, which is dependent on vitamin-D-binding protein concentrations and genotype, is approximately 15 days and serves as a clinical measure of vitamin-D status, whereas the half-life of calcitriol is much shorter (5–15 h), therefore, its local production is advantageous (11, 17).

## The Role of Vitamin-D in Immunity and Immunomodulation

**Figure 2:**
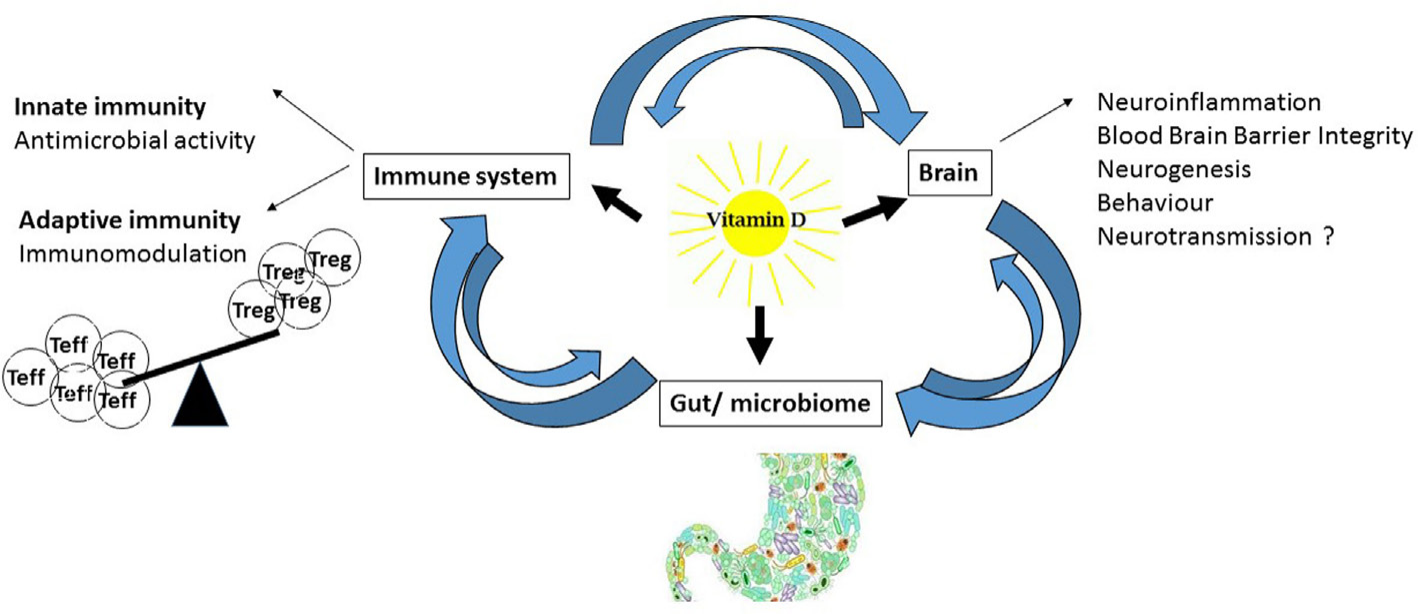
**Effects of vitamin-D: schematic diagram of vitamin-D effects on the immune system, brain, and gut**.

Vitamin-D is known for its skeletal effects; however, in this review, we will focus on non-classical vitamin-D physiology and its involvement in immunity and inflammation (Figure 2). Two major observations link vitamin-D to immunity. First, most proliferating immune cells express the vitamin-D receptor (VDR) for active vitamin-D. The VDR is expressed in immune cells of the adaptive and innate immune system, such as T-cells, B-cells, monocytes, macrophages, dendritic cells (DCs), and neutrophils (18). Additionally, immune cells exhibit an active vitamin-D metabolism with the expression of the rate-limiting enzyme for vitamin-D synthesis, 1α-hydroxylase (CYP27B1) (19). Immune cells are, therefore, able to synthesize and secrete vitamin-D in both an autocrine and paracrine fashion, indicating that vitamin-D plays an important role in the immune system, where it affects antigen presentation, innate immunity, and T-cell activation, differentiation, and migration (19, 20).

## The Impact of Vitamin-D on Innate Immune Responses and Antimicrobial Responses

Genome-wide analyses and associated *ex vivo* and *in vitro* experiments have clearly demonstrated the importance of vitamin-D in orchestrating innate immune responses and maintaining optimal antibacterial responses in humans. Our innate immune system recognizes pathogen-associated molecular patterns (PAMPs) with the help of the so-called pattern recognition receptors including toll-like receptors (TLRs) to mount successful immune responses for the successful eradication of pathogens. A role for vitamin-D metabolism and signaling in innate immunity was provided by a genome-wide approach, which showed that the macrophage response to *Mycobacterium tuberculosis* involved an endogenous, intracrine vitamin-D system. Exposure to a TLR-2-interacting PAMP induced the expression of both CYP27B1 and VDR in macrophages (21). Furthermore, expression of antimicrobial proteins (cathelicidin, β-defensin-2, hepcidin antibacterial protein) can be induced by vitamin-D in macrophages upon pathogen encounter (22). The complex induction involved cooperation between VDR and NF-κB, which is the major transcription factor that regulates genes responsible for both the innate and adaptive immune responses but is also implicated in neuronal plasticity and memory (23). The innate immune response comprises a pronounced inflammatory component, and vitamin-D counteracted these events by promoting hyporesponsiveness to PAMPs *via* downregulation of TLRs on monocytes (24). In addition, other vitamin-D-mediated innate immune functions comprise the regulation of the nitric oxide pathway, iron metabolism, and autophagy, an intracellular degradation system thought to play an important role in neurodegeneration (19, 25).

## The Impact of Vitamin-D on Antigen Presentation and Adaptive Immune Responses

Antigen-presenting cells are important players of our immune response and pivotal in priming and orchestrating adaptive immune responses. The function of monocytes, macrophages, and DCs can be modulated by vitamin-D as they exhibit an active intracrine vitamin-D system and express VDR and CYP27B1. Vitamin-D was also able to influence the differentiation of DCs (26). Furthermore, *in vitro* addition of vitamin-D to antigen-presenting cells inhibited the surface expression of antigens by major histocompatibility complex (MHC) class II and its costimulatory molecules, leading to reduced T-cell stimulatory capacity (27). Several studies highlighted a role for vitamin-D as inhibitor of T- and B-cell proliferation (28); however, it has become increasingly clear that the prominent effects of vitamin-D involve the modulation of the T-cell phenotype of CD8^+^ cytotoxic T-cells and T-helper (Th) cells. Several T-cell subgroups have been characterized according to their distinct cytokine profiles. Interestingly, vitamin-D directly exerted its immunomodulatory effects on T lymphocytes by inhibiting the production of pro-inflammatory Th1 cytokines (IL-2, IFN-γ, and TNF-α, considered to be the key mediators in graft rejection and autoimmune diseases) and stimulated the production of anti-inflammatory Th2 cytokines (IL-4, IL-5, and IL-10), which have immunoregulatory functions (29). Vitamin-D also drove immunomodulation by suppressing inflammatory IL-17-expressing Th17 cells and promoted the production of regulatory T-cells (Treg) (30). Recent studies showed that Treg function correlated with serum concentration of vitamin-D in multiple sclerosis (MS) patients (31). Of interest is also the capacity of vitamin-D to influence T-cell homing (32).

The action of vitamin-D on cellular immune responses has been the focus of much research; however, not much is known on the effect of vitamin-D on B-cell homeostasis. However, it is known that the effect is not restricted to their IgG-producing capacity. Vitamin-D suppressed the differentiation of plasma cells and class-switched memory cells and regulated B-cell IL-10 production (33, 34).

## Vitamin-D and the Microbiome

The gut is the largest immune organ in the human body, and the gut microbiome plays an important role in health and disease. The gut microbiota is thought to communicate with the brain and regulate central nervous system (CNS) homeostasis through immune, vagal, and metabolic pathways. Alterations in its composition have been implicated in a wide range of neurological and psychiatric conditions including MS, schizophrenia, and autism, which have been reviewed in detail elsewhere (35, 36). The effect of vitamin-D on the microbiome, and *vice versa*, is less well researched, but a recent genome-wide association study (GWAS) showed that variation in the VDR influenced the composition of gut microbiota (37). This finding and the importance of the gut–brain axis in shaping behavior and brain development warrants further research as it will open up a new and exciting line of investigation.

## Vitamin-D and the Brain

An increasing body of evidence suggests that vitamin-D is an important player in mature brain function and brain ontogeny (38). The effects of gestational developmental vitamin-D deficiency in adult offspring led to persistent effects on brain anatomy, neurochemistry and function, where it impacted on neuronal differentiation, axonal connectivity, and dopamine ontogeny (39). Furthermore, vitamin-D influenced neural stem cells proliferation, survival, and neuron/oligodendrocyte differentiation supporting its remyelinating and neuroprotective effects (40). The effect of hypovitaminosis-D on synaptic imbalances warrants further study.

The VDR was found expressed in the human and rodent brain, and its widespread distribution suggests that vitamin-D may have autocrine/paracrine properties. The strongest immunohistochemical staining for the VDR and 1α-hydroxylase was found in hypothalamus and in the large neurons within the substantia nigra (16). Earlier studies using radiolabeled vitamin-D showed accumulation in nuclei of neurons, which suggested its role in regulating the production of several aminergic and peptidergic messengers and influencing the activity of certain sensory, motor, and endocrine–autonomic systems (41). The receptor was also found expressed in oligodendrocyte-like cells, human leukocyte antigen (HLA)-positive microglia, and glial fibrillary acidic protein-positive astrocytes (42).

Microglia cells are key players of the immune system in the CNS and play an important role in brain infections and brain development. Their activation interferes with neuronal survival by increasing oxidative stress and decreasing neurotropic support and has been linked to MS (43), schizophrenia (44, 45), and autism (46). As microglia cells play a pivotal role in neuroinflammation and neurodegeneration, downregulation of their pro-inflammatory cytokine production and release of free radicals by vitamin-D may be neuroprotective (47).

## Vitamin-D in MS

Multiple sclerosis is an inflammatory, demyelinating disease of the CNS characterized by myelin loss, inflammatory lesions, and varying degrees of axonal pathology. It is a leading cause of disability in young adults, found to be more prevalent in woman, and affects 2.5 million people worldwide. The etiology of MS is still unknown, but autoimmune processes are thought to play an important role (48).

Susceptibility depends on genetic and environmental risk factors and their interactions (49). The study of environmental risk factors is of great interest as they can potentially be modulated, in contrast to the genetic susceptibility. This may offer exciting innovative strategies for disease prevention and intervention. Several risk factors are the current focus in MS research such as hypovitaminosis-D, viral infections (Epstein–Barr virus, human herpes virus-6, and human endogenous retroviruses), smoking, and the microbiome (43, 49–54). Interestingly, recent studies showed an association between vitamin-D and EBV status, which may highlight a role of vitamin-D in control of persistent EBV infection (51). In this review, we will focus on hypovitaminosis-D and summarize the latest findings in MS.

## Vitamin-D Studies in MS Patients

There is a substantial body of evidence on the role of vitamin-D in the development of MS. Two important prospective studies showed a protective effect of vitamin-D in MS. A nested case–control study in US military personnel reported that high serum concentrations of 25-hydroxycholecalciferol correlated with decreased MS risk (55). A more recent prospective study confirmed these findings and reported that levels of vitamin-D over 75 nmol/L were associated with a decreased MS risk (56).

Several observational studies have consistently shown an association of low serum levels of vitamin-D with increased MS risk and supported the findings from the prospective studies. Vitamin-D intake was found to moderately decrease the risk of MS in a large prospective study (*n* = 187,563) (57). In addition, the influence of vitamin-D on the disease course of MS is equally strong. Vitamin-D status correlated inversely with exacerbation risk in relapsing-remitting MS and suggested a beneficial effect on MS disease activity (58, 59). This effect was also found in patients on interferon-β treatment, where the lowest rate of new lesions was found in patients with vitamin-D levels over 100 nmol/L (60, 61). Of interest is also a potential role for vitamin-D in the conversion from clinically isolated syndrome, a first event suggestive of MS, to clinically definite MS. Low vitamin-D levels early in the disease course may predict higher risk of conversion to clinically definite MS (62).

Additional studies showed that genetic effects on vitamin-D pathways may also contribute to MS risk. Two recent Mendelian randomization studies evaluated whether genetically lowered vitamin-D levels influenced the risk of MS. The first study identified four single-nucleotide polymorphisms (SNPs), which were in or near genes strongly implicated in vitamin-D metabolism. Consecutive Mendelian randomization studies showed that genetically lowered 25(OH)D levels were strongly associated with increased susceptibility to MS (63). These findings were confirmed in a recent study, which reported strong evidence of a causal effect of low serum 25(OH)D on MS risk that is independent of established environmental risk factors and not subject to reverse causality (64). Interestingly, MS risk was found to be associated with the gene encoding the enzyme that activates vitamin-D (CYP27B1) and the genetic variant rs703842 in CYP27B1 in Caucasians (65, 66). Additional findings highlight that vitamin-D may also be able to regulate genes of the immune system that play a role in MS development. Molecular studies showed that MS associated loci were enriched for VDR-binding sites, including the promoter region HLA-DRB1 (67, 68).

Several vitamin-D supplementation trials are currently underway either as stand-alone or add-on therapy to disease modifying treatment. The largest study so far is the SOLAR study, which enrolled 229 interferon-β-treated MS patients with a 25-hydroxyvitamin-D plasma concentration below 150 nmol/L. This double-blind placebo-controlled study of high-dose oral cholecalciferol oil (14,000 IU/day) showed intriguing results. The primary endpoint “no evidence of disease activity” was not improved by vitamin-D supplementation; however, the secondary endpoint showed a 32% reduction in the number of new combined unique active lesions in the cholecalciferol group. Furthermore, there was a trend toward absence of new T1 hypointense lesions in vitamin-D-supplemented patients, which became significant in those aged 18–30 years. Therefore, the results support the notion that vitamin-D supplementation is a safe and an effective add-on treatment in MS patients on β-interferon (69). The SOLAR study results support the findings of an earlier smaller Finish randomized trial in which patients receiving 20,000 IU vitamin-D_3_ per week had better MRI outcomes than those receiving placebo (70). This study and observational small studies support the notion that similar benefit can be obtained from much lower levels of vitamin-D supplementation (equivalent to about 3,000–4,000 IU/day) and supraphysiological doses (14,000 IU/day) like in the SOLAR study are not needed.

## Vitamin-D in Schizophrenia

In the second part of our review, we will discuss the role of vitamin-D in schizophrenia. Schizophrenia is a debilitating psychotic disorder that develops most commonly during the late adolescent to early adulthood period across both genders, with females having a later age of onset. It is debilitating in the sense that the sufferer’s ability to function normally in society is heavily impaired by a range of positive (hallucinations and delusions), negative (avolition, anhedonia, and alogia), and cognitive symptoms (71).

Schizophrenia lifetime prevalence is about 1% of the general population, meaning millions of people worldwide suffer from the disease (72). However, in some ways, this statistic fails to represent the true number of individuals affected by this mental illness, as friends and family members will also be heavily impacted.

Schizophrenia manifests as a mixture of cognitive, negative, and positive symptoms. Positive symptoms refer to psychosis, such as hallucinations and delusions, while negative and cognitive symptoms refer more to impairments in emotional, social, and intellectual functioning. Distinguishing between negative and cognitive symptoms is difficult, but they should be viewed as independent targets for intervention (73). However, both are linked closely and non-respondent to antipsychotics, making their treatment difficult (74).

An important observation, supporting the notion of a biological disease and, indeed, the connection between infection, immune responses, and psychosis, was made by Julius Wagner-Jauregg as early as 1883 (75). A relationship between fever and “madness” had been postulated over centuries; in clinical experimentation at the Vienna asylum, Wagner-Jauregg injected patients suffering from tertiary syphilis or dementia paralytica with potent immunostimulators such as tuberculin and malaria. Some patients made remarkable recoveries—far more than without treatment. Although nowadays these experiments would be ethically forbidden for good reasons, they marked a paradigm shift in psychiatry and Wagner-Jauregg received the Noble prize in 1927 for his work on “pyrotherapy” (fever therapy). The British and American clinicians W. L. Templeton and Leland Hinsie went on to try fever treatment therapy on schizophrenic patients with observed improvement in some patients, however, not permanent. This line of investigation was given up due to the danger of the malaria treatment and the transient nature of improvement. Looking back at these experiments with our knowledge of the twenty-first century immunology, one observes that these may have been the first findings signposting the role of the immune system in schizophrenia.

There appears to be a diffuse non-specific activation of the immune system in schizophrenia (76). Further evidence of this comes from genome-wide associations mapping to the MHC region in schizophrenia susceptibility and immune cells involved in adaptive immunity (CD19 and CD20 B-lymphocytes) (77, 78). Notably, in 2014, in what was at the time the largest genetic study of mental illness, the researcher identified 108 loci associated with schizophrenia. Interestingly, recent GWASs have now robustly identified immune-related SNPs linked to schizophrenia. Recent cross genomic studies, which addressed the common architecture between schizophrenia and those of other psychiatric and non-psychiatric traits, revealed links between schizophrenia and MS (79). A significant genetic overlap was found between schizophrenia and MS mainly within the MHC. This study demonstrated the involvement of the same HLA alleles in MS and schizophrenia, but with an opposite directionality of effect. Intriguingly, recent population-based studies found that several psychiatric comorbidities including schizophrenia, anxiety, depression, and bipolar disorder were more common in MS population than in a matched control cohort (79, 80), as were white matter changes and myelin-related dysfunction in schizophrenia (81).

This work suggests that a subgroup of patients with schizophrenia may demonstrate aspects of an autoimmune process. Interestingly, the elimination of autoantibodies against neuronal cell surface proteins by immunotherapy has led to symptomatic improvement in some cases of first-episode psychosis (82). One putative environmental risk factor for schizophrenia is infection. Early childhood infections of the brain increase the risk ~5-fold (83). Even during pregnancy, particularly the second trimester, maternal infections correlated with an increased risk to the offspring later in life (84). Injecting pregnant mice with synthetic double-stranded DNA poly I:C, to mimic viral infections/interferon responses, and lipopolysaccharide, a highly inflammatory component of bacterial cell walls, elicits morphological and behavioral changes characteristic of the brain in schizophrenia (85); however, the underlying mechanisms are not fully understood.

The autoimmune hypothesis is also strengthened by the finding of increased autoimmune disease in relatives of schizophrenic patients and the inverse relationship of schizophrenia with rheumatoid arthritis and connective tissue diseases (86). Notably, a very recent study singles out the gene C4, a component of the intricate complement system that works together with the immune response to regulate immune tolerance, autoimmunity, and anti-pathogen responses, as the strongest genetic risk factor for schizophrenia (87). These findings have lent additional support to theories regarding immunological dysregulation as an underlying cause of schizophrenia. However, there is no evidence for a direct link between vitamin-D and C4 levels.

The hypothesis of an active immune/inflammatory component, which lends support to the “mild encephalitis hypothesis” (88) in at least a subgroup of schizophrenia patients, is of great interest and might prompt novel preventive or therapeutic strategies, such as immunomodulation and/or anti-inflammatory drugs. A recent meta-analysis of anti-inflammatory medications in the management of treatment-resistant schizophrenia showed therapeutic effects of fish oils, *N*-acetyl-cysteine, and estradiol (89).

## Vitamin-D Studies in Schizophrenia Patients

Several lines of evidence support a role for vitamin-D deficiency in the risk for schizophrenia. Epidemiological data suggest that schizophrenia is more common in those born in winter and spring and its prevalence also rises with increasing latitude (90). These findings, added to the evidence that dark-skinned minority groups in cold countries have a greater risk of schizophrenia (91), have led to the hypothesis that low vitamin-D (especially during early life) may be implicated in the genesis of schizophrenia (92). A study based on Danish neonatal dried blood spots supported this hypothesis (93), compared with neonates in the fourth quintile (vitamin-D_3_ concentrations between 40.5 and 50.9 nmol/L), those in each of the lower three quintiles had a significantly increased risk of schizophrenia (twofold elevated risk).

Patients with psychosis have lower levels of vitamin-D than matched controls, even at the first presentation with psychosis (94–96). A mini-meta-analysis confirmed that schizophrenia patients have lower vitamin-D levels than healthy controls with a medium effect size (97). A systematic review (based on seven studies) has confirmed that those with psychosis are significantly more likely to have low concentrations of vitamin-D (98). Moreover, looking at specific schizophrenia symptomatology, levels of vitamin-D have been shown in some studies to inversely correlate with depression and negative symptoms, in patients with psychosis, controlling for other contributors (99).

Low vitamin-D may have also detrimental effects on brain development. The Dutch Hunger Winter and Chinese Famine studies have suggested a role for hypovitaminosis-D in the development of schizophrenia. However, findings regarding vitamin-D deficiency and its link to psychosis have the potential to be confounded by factors such as other nutrient deficiencies and ethnicity/skin tone. As a result, investigations around this association with schizophrenia are mainly supported by the more clear evidence of vitamin-D as a protective neuro-immunomodulator, which strongly suggests that hypovitaminosis-D during development would have profound effects on offspring outcome (100).

Concerning the impact of low vitamin-D on the adolescent and adult brain, a study based on a UK birth cohort (*n* = 3,182) found an association between low vitamin-D among children with a mean age of nine years and an increased risk of later psychotic-like symptoms during adolescence (mean age 14 years) (101). Diet also appears to be important—a large population-based study of Swedish women (*n* = 33,623) reported a significantly greater risk of psychotic-like experiences in those with low vitamin-D intake (102). Thus, the evidence suggests that low vitamin-D not only disrupts early brain development but may also compromise later periods of brain growth and maturation.

A prospective Finnish birth cohort study looked at one way of addressing this risk and found that vitamin-D supplementation in males during the first year of life resulted in a reduced risk of them later developing schizophrenia (103).

It is therefore plausible that vitamin-D may reduce inflammation and enhance resilience to neurobiological or pathogen-induced insults, which might increase risk of schizophrenia. Indeed, two cross-sectional studies have reported that vitamin-D is inversely associated with levels of C-reactive protein, a marker of inflammation, in psychosis (96, 104).

Despite all this circumstantial evidence, a recent Mendelian causation study (105), looking at SNPs associated with serum vitamin-D and schizophrenia in 34,241 schizophrenia cases and 45,604 controls, found no evidence for causal effect of vitamin-D on the risk for schizophrenia. Moreover, currently, there is no evidence from randomized controlled trials of vitamin-D supplementation in the relevant populations, although a trial is underway. Those randomized controlled trials are needed to confirm the effect of vitamin-D supplementation on inflammation in patients with schizophrenia.

## Vitamin-D in Autism Spectrum Disorder (ASD)

Autism spectrum disorders are a heterogeneous group of complex neurodevelopmental disorders that undermine optimal brain development. A recent surveillance study identified 1 in 68 children (1 in 42 boys and 1 in 189 girls) as having ASD. Recent data collated for ASD suggest that the cost is at least £32 billion a year (106).

While ASD is currently diagnosed on the basis of abnormalities in social communication and repetitive behaviors, it is increasingly being recognized as a whole-body disorder. The core behavioral characteristics such as altered communication and social skills, cognitive and learning deficits, and stereotypic behaviors are being intrinsically linked to complex biological processes.

Increasing evidence suggests that altered immune responses in ASD may be related to the severity of behavioral impairment and other developmental outcomes (107). Abnormal cytokine profiles have been described with elevated levels of pro-inflammatory cytokines (e.g., IL-6, IL-8, IL1β, IFN-γ, and eotaxin) (108). Several *postmortem* and neuroimaging studies have found chronic neuroinflammatory processes such as microglial activation in the CNS (109, 110). It has been suggested that this chronic immune activation could be a response to an early autoimmune attack on the brain by mother-to-fetus transfer of autoantibodies and/or maternal infection (111–114). Research into antibody-mediated CNS disorders may help identify a subgroup of patients with antibody-mediated illness, which may be relevant to autism and schizophrenia (115). Findings from animal models in ASD point toward inflammatory processes; and anti-inflammatory/immune-modulating drugs in ASD have been trialed (116). It is now well established that individuals with autism have much higher than expected rates of a range of comorbidities, which support dysregulation of immune mechanisms, inflammation, and a potentially altered gut–brain axis and resemble findings in other inflammatory and autoimmune diseases (117). Autoimmune and gastrointestinal problems are often present in ASD, and nutritional approaches have become widely used in managing ASD (118).

Autism spectrum disorder is an extremely heterogeneous disorder. It is suggested that the origin of ASD influences the phenotype—thus, e.g., ASD caused by maternal infection during pregnancy may trigger a different set of symptoms than more genetically driven forms of ASD or combinations of environmental (pollution, neurotoxins, etc.) and genetic factors (118). In addition, gender seems to play an important role as girls with ASD often present with milder social and communicative symptoms, relatively intact symbolic play skills and fewer obsessional interests (119). Ecological studies observed the correlation between the number of ASD cases and a number of environmental factors such as latitude, season of birth, mother’s skin type, and the climate, implicating a possible role for vitamin-D in ASD (120).

## Vitamin-D Studies in ASD

Several studies found lower vitamin-D levels in children with autism compared to their siblings, parents, and non-family controls (17, 121). Low vitamin-D levels were already present at birth in children later diagnosed with ASD but not in their healthy siblings (122). Subsequent research demonstrated that the vitamin-D status of mothers corresponded with their offspring’s vitamin-D status at birth. Low levels of vitamin-D during pregnancy impacted negatively on the cognitive status, early development, and ASD diagnosis (123).

Two studies have addressed the impact of vitamin-D supplementation on ASD. One found improved core symptoms in children supplemented with pharmacological doses of vitamin-D (124). Furthermore, a preliminary study found that vitamin-D supplementation during pregnancy and early childhood decreased the occurrence of ASD in siblings (125). However, optimal vitamin-D dosage and levels are not yet determined, and proper randomized trials are needed.

Several studies looked at vitamin-D-specific gene variants and the risk of ASD. Recently, paternal and child genetic abnormalities in vitamin-D metabolism in ASD were reported (126). Notably, paternal VDR *Taq*I homozygous variant genotype and VDR *Bsm*I and offspring’s GC AA-genotype/A-allele were associated with ASD, whereas offspring’s CYP2R1 AA-genotype was significantly associated with decreased risk of ASD.

Further support for a role of vitamin-D comes from a recent study, which reported association between polymorphisms in the VDR gene and vitamin-D levels in ASD children (127). Vitamin-D levels were influenced by *Fok*I polymorphisms and haplotype GTTT (*Bsm*I/*Taq*I/*Fok*I). Interestingly, this polymorphism resulted in compensatory higher vitamin-D levels due to lower VDR activity in ASD akin to the findings reported in MS (127, 128).

Of particular interest is the observed strong gender bias in ASD (four males:one female), which may be suggestive of abnormalities in steroid metabolism, which comprises the hormones cortisol, testosterone, estrogens, progesterone, and vitamin-D. Indeed, ASD children have significantly higher levels of a number of C21 and C19 steroid hormones, especially androgens (129, 130). Altered steroid hormone levels have also been identified in children with Smith–Lemli–Opitz syndrome (SLOS), which carries a comorbid ASD risk of 50–86% (131). Mutations in the DHCR7 gene that codes for the enzyme 3β–hydroxysterol-Δ(7)-reductase, the catalyst for the final step in cholesterol biosynthesis (132), lead to hypo-cholesterolemia and often higher levels of 7-dehydrocholesterol in SLOS patients. Dysregulation of the steroid metabolome, in synergy with genetic predisposition and other environmental risk factors (e.g., methylation, maternal infection, neurotoxins and other chemicals, premature birth, paternal age), may act as a potential risk factor for the development of ASD, schizophrenia, and other mental disorder with hypovitaminosis-D being one of the possible hallmarks (133).

Autism spectrum disorder research and diagnosis would benefit from greater analysis of metabolic markers and genetic polymorphisms, to aid patient stratification and identify therapeutically relevant biomarkers to inform diagnosis, prevention, and treatment strategies. Collaborations between geneticists, immunologists, steroid chemists, endocrinologists, nutritionists, psychiatrists, and psychologists will be needed to decipher the complex pathophysiology of ASD.

## Conclusion

Multiple sclerosis, schizophrenia, and autism are multifactorial disorders caused by the effects of multiple genes in combination with environmental factors. As environmental risk factors are modifiable—in contrast to genetic susceptibility—they offer potential strategies for intervention and prevention. Great efforts are being made in identifying risk factors in these conditions and vitamin-D is one of the culprits, with most evidence in MS, where hypovitaminosis-D seems to contribute to disease activity and vitamin-D supplementation studies have shown some promise.

There is also some evidence, albeit less clear, that hypovitaminosis-D may act as risk factors for schizophrenia and autism, and further research and longitudinal studies are needed. Inflammatory responses appear to play a significant role in the etiology of both schizophrenia and autism. Whether and how vitamin-D contributes to the pathophysiology of these conditions is unknown. Further insight into the role of vitamin-D, in schizophrenia and autism, especially as it relates to the immune system, inflammation, and neuroprotection, will help shed light on the underlying pathophysiology of these conditions and may aid the design of better treatment strategies for the twenty-first century.

## Author Contributions

U-CM, AK, and EK helped in drafting the manuscript, and U-CM, AK, EK, and FG helped in reviewing the manuscript.

## Conflict of Interest Statement

Ute-Christiane Meier has a patent pending: “Biomarker for inflammatory response.” The remaining authors declare that the research was conducted in the absence of any commercial or financial relationships that could be construed as a potential conflict of interest.
